# A qualitative analysis of barriers and facilitators to reducing sedentary time in adults with chronic low back pain

**DOI:** 10.1186/s12889-021-10238-5

**Published:** 2021-01-26

**Authors:** Jeni E. Lansing, Laura D. Ellingson, Kathryn J. DeShaw, Gabriel Cruz-Maldonado, Tera R. Hurt, Jacob D. Meyer

**Affiliations:** 1 Department of Kinesiology, Iowa State University, Ames, IA USA; 2 Division of Health and Exercise Science, Western Oregon University, Monmouth, OR USA; 3 Department of Kinesiology, Loras College, Dubuque, IA USA; 4 Department of Human Development and Family Studies, Iowa State University, Ames, IA USA

**Keywords:** Sedentary behavior, Chronic low back pain, Qualitative analysis

## Abstract

**Background:**

Sedentary time (SED) is associated with many detrimental health outcomes, yet little is known about what factors influence one’s ability to reduce SED. Even less is known about these factors in specific patient populations for whom high levels of SED may influence symptoms, such as those with chronic low back pain (cLBP). The purpose of this study was to qualitatively explore participants’ perceptions of factors that influenced their ability to reduce SED across an 8-week intervention to reduce SED in adults with cLBP and elevated depressive symptoms.

**Methods:**

Three months after a theory-based intervention to break up and reduce sitting, semi-structured interviews explored factors that influenced reducing SED. Three researchers independently coded each conversation. Codes were charted and mapped with participants reviewing their own transcripts and the merged codes. The research team then defined key themes. Factors that were perceived to either facilitate behavior change or acted as barriers were identified and thematized as positive or negative determinants.

**Results:**

Common barriers for reducing SED included environmental constraints, opposing social norms, and productivity; these barriers were frequently encountered in the workplace. Common facilitators for reducing SED included habit development, self-monitoring tools, restructuring the physical environment, and social accountability. Notably, back pain was not a frequently reported barrier or facilitator for reducing SED.

**Conclusion:**

This sample of patients with cLBP and elevated depressive symptoms had similar determinants for reducing SED as previously reported in non-patient populations and did not appear to need strategies specific to dealing with chronic pain. Since work-related social norms and environmental factors were perceived as significant barriers to sitting less, workplace interventions that provide standing desks, offer standing meetings rooms, and/or institution-wide standing breaks may help reduce SED at work. The use of an activity monitor with sitting reminders and education regarding how to use the reminders as external cues to develop new sitting habits may also aid in adoption and adherence to this behavior change across settings. Developing coping plans and restructuring physical environments were perceived as successful strategies for overcoming social and environmental barriers. Future interventions targeting SED reductions may benefit from incorporating these strategies.

**Supplementary Information:**

The online version contains supplementary material available at 10.1186/s12889-021-10238-5.

## Background

Recent data show that US adults are spending much of their time sedentary [1]. Sedentary behaviors are defined as any waking behavior with a low energy expenditure, while in a sitting, reclining, or lying posture [2]. High sedentary time (SED) is associated with many health conditions including chronic pain [3–5]. To effectively target reducing SED, a better understanding of factors that influence SED is needed. While the research on determinants for increasing physical activity is vast across a variety of populations [6], factors that influence reducing SED are less understood.

To date, research on determinants for reducing SED has focused primarily on demographic factors and correlates, rather than qualitatively asking individuals about factors they believe influence their sitting behaviors and their potential for change [7–9]. Further, the majority of qualitative studies evaluating SED have focused on healthy and/or older adults [10, 11] who may have different perspectives than clinical populations, such as adults with chronic low back pain (cLBP). Chronic low back pain is among the top five leading causes of disability worldwide and these patients report higher rates of depression [12, 13]. Pain and depressive symptoms are both common and may influence perceived barriers and facilitators for changing sitting behavior but no studies have been published that examine determinants for decreasing SED in this population.

Reducing SED may be especially relevant, as high levels of SED are a risk factor for the development of cLBP [4, 5] and decreasing SED has been associated with improvements in back pain [14, 15]. To date, sedentary interventions have employed several behavior change strategies including habit theory and mHealth [14, 15]. Theoretically, these strategies may be particularly helpful for reducing SED by modifying individual micro-environments through creating reminders (e.g. cues from wearable technology) to stand and move, providing real-time feedback of the behavior, and/or generating friction against sitting [16, 17]. However, the utility of these approaches from the participant’s point of view is unclear as no studies have explored *patient perspectives* of the effectiveness of strategies like habit development and wearable technology *following* their attempts to implement these strategies to reduce SED.

Qualitative insight into the challenges that participants encounter during SED interventions may provide valuable information about barriers and facilitators to decreasing SED. This information can then be used for developing interventions that are feasible from the patient perspective, lead to sustained SED behavior change, and ideally result in symptom improvement in individuals with cLBP. Therefore, the purpose of this study was to qualitatively examine patient-reported factors influencing their ability to reduce SED 3-months after an 8-week SED intervention for adults with cLBP.

## Methods

### Participants

Data was collected by questionnaire and participants were selected using a convenience sampling procedure from participants who completed a theory-informed intervention targeting reductions in SED and improvements in symptoms of cLBP (ClinicalTrials.gov Identifier: NCT04257539). Participants for this study were included if they were: between the ages 25–60, not taking immunomodulatory medication, on stable medication regimen over the past 8 weeks and willing to maintain current medications, not pregnant or planning on becoming pregnant, willing to wear a Fitbit monitor for 3 months, not currently using a commercial activity monitor with an idle alert feature, and able to regularly access the internet or a smartphone. Participants were excluded if: they had injuries or health conditions that prevented them from safely participating in physical activity, did not have chronic low back pain defined as experiencing symptoms every day or nearly every day for longer than 3 months, or not experiencing elevated symptoms of depression (Patient Health Questionnaire-9 ≤ 5).

### Procedures

Participants (*n* = 20) randomized to the intervention group completed a pre-intervention visit, an 8-week SED intervention, and a post-intervention visit. All visits were face-to-face and completed by the same research team member (JL). During the pre/post intervention visits, data regarding cLBP symptoms, sitting habits, and SED were collected. The SED intervention included provision of a commercial activity monitor (Fitbit Alta, Fitbit Inc., San Francisco, CA) and two sessions with a health coach trained in Motivational Interviewing (researcher JL), occurring immediately before the intervention period and at 4 weeks. During the first session, participants discussed their current sitting behaviors informed by data from 7 days of wearing an activPAL3 (PAL Technologies, Glasgow, Scotland, UK). These data revealed information regarding the time(s) of day and durations that most sitting occurred over the previous week. Additionally, intervention study goals, health risks of prolonged sitting, strategies for changing sitting habits and developing new habits of sitting less (e.g. implementation of internal and external cues), and personal motivations for changing this behavior were discussed.

During the intervention, participants were instructed to reduce total SED per day and break up prolonged sedentary periods. To facilitate breaking up prolonged sedentary periods, they were specifically instructed to accumulate 250 or more steps in 8 or more hours of the day using the idle alert on the activity monitor as a reminder and external cue. Compliance to this 8 h/day goal was monitored weekly by the study team and participants were contacted and reminded of these intervention requirements if they were not meeting them. Three months after the intervention, after follow-up data were collected, participants completed a 20–30 min semi-structured phone interview with their health coach (JL) about factors that influenced their ability to reduce SED during the intervention. Qualitative procedures were added after 9 participants completed the follow-up period, so interviews were conducted with 11 total participants. Audio from these conversations were recorded using a recording platform (Zoom Local Recorder, Zoom Video Communications, San Jose, CA) and stored for transcription. All participants were in private rooms either at home or work during the interviews, with no one else present at the time. Each participant completed one interview (i.e. no repeat interviews). An initial analysis sample and stopping criteria were not pre-determined to establish data saturation due to convenience sampling from the SED intervention (i.e. 11 eligible participants from larger intervention trial). Nonetheless, all coders agreed no new codes or ideas emerged after interview nine.

### Measures

#### Qualitative interview

Semi-structured interviews were used to collect data for this study. A standard set of questions was used to guide conversations, with the interviewer modifying the interview with follow-up questions and/or probes based on participant responses (see interview script in Supplementary file 1). The questions chosen for this specific interview were selected based on study objectives and previous qualitative semi-structured interviews investigating determinants of sedentary time [10]. Experts in exercise, health, and social psychology reviewed the questions to ensure appropriate language was used and questions aligned with study objectives.

#### Descriptive health, activity, and habit questionnaires

Descriptive data used in this study were collected with several measures before and after the intervention. A baseline demographic questionnaire was administered to characterize the sample, with respect to age, sex, race, education, marital status, occupational status, household income. The Minimal Data Set for Chronic Low Back Pain was used to quantify symptoms of cLBP and impact on daily life, as recommended in the Report of the NIH Task Force on Research Standards for Chronic Low Back Pain [18]. Scores on this questionnaire range from 8 (mild impact) to 50 (severe impact). Depressive symptoms were assessed using the psychometrically strong (Cronbach’s α =0.86–0.89) Patient Health Questionnaire-9 (PHQ-9), with total scores (ranging 0–27) categorized 0–4, 5–9, 10–14, 15–19, and 20–27 as minimal, mild, moderate, moderately severe, and severe depression, respectively [19]. The SIT-Q-7d was used to assess self-reported SED over 7 days in different domains, including eating meals, occupational, transportation, household/leisure activities, and screen-based activities [20]. This measure has demonstrated adequate test-retest reliability (ICC = 0.65) and convergent validity, moderately correlated (*p* = 0.53) with the SIT-Q and 7 Day Activity Diary [20]. Habit development was assessed using the Self-Reported Habit Index (SRHI), consisting of 12 items scored on a Likert scale with anchors ranging from strongly disagree (1) to strongly agree (7), with lower scores indicating weak habits and higher scores indicating strong habits (total scores ranging 7–84) [21]. The behaviors included in this study were “frequently standing up,” “breaking up long bouts of sedentary activity,” and “sitting when I could stand,” with participants responding to all 12 items for each of these behavior prompts (i.e., 3 prompts with 12 items each for a total of 36 responses). The SRHI has demonstrated high test-retest reliability, with a coefficient of 0.89–0.91 [21].

#### Monitor-assessed activity

Physical activity and SED were assessed using activPAL3 activity monitors. The monitors were worn for 7 days (24 h/day) one week prior to the start of the intervention and during the final week of the intervention. These are small triaxial accelerometers that are worn on the thigh and classify wear time as sedentary, upright or stepping. PALanalysis (version 8.10.8.76), the manufacturer’s associated software, provided data and graphics of total SED and different bout lengths of SED each day the monitor was worn, which were shared with participants during their first health coach session to educate them regarding their current sitting behaviors. In previous studies, the activPAL3 has demonstrated excellent validity compared to direct observation for assessing SED and physical activity in free-living conditions [22].

### Analysis

Descriptive statistics were calculated regarding participant demographic characteristics and from pre/post intervention data to characterize the sample and provide supplementary information regarding changes in habit development, self-reported SED, and monitor-assessed SED pre and post intervention.

A thematic analysis was used to identify key themes from the structured interviews, following processes recommended by Miles, Huberman, and Salacia (2019) [23]. Each interview was transcribed and independently cross-checked for errors. Three researchers (JL, KD, and GC; see Supplementary file 2 for further detail) were responsible for coding the conversations, jointly creating a deductive codebook (see Supplementary file 3) based on theoretical constructs used in the SED intervention (e.g. cues: habit theory; self-monitoring: mHealth) and previous literature of determinants of SED (e.g. social norms, environmental constraints, physical discomfort) to guide initial coding.

Following this, coders familiarized themselves with the data by reading each transcript several times. Coders then separately identified codes for each conversation, while refining codes and definitions in the codebook, jotting, and writing memos. After coding was complete, each participant was sent their coded transcription (with merged comments from all 3 coders) to review and provide corrections and/or clarification on their comments and researcher codes. Feedback from participants was then discussed by all three coders until a revised code was agreed upon.

Next, each interview was indexed, in which relevant text sections were copied and sorted into corresponding codes (i.e. charted). Subsequently, the coders met to review and compare coding and establish final thematic categories and associated definitions. For the purposes of this study, a thematic category was one that illustrates something important about the data with specific regard to the research question and is a patterned response among multiple participants [24]. Summaries of the themes were mapped by creating a matrix to define each category and provide participant quotes that exemplified each theme. Then, a network display was created to explore potential relationships among themes. Finally, the research team met to discuss and interpret the findings.

## Results

Participant demographic information is provided in Table 1. No participants refused to participate or withdraw from the study. Overall, men and women were similarly represented, and the sample was predominately white. Most participants worked full time in a variety of occupations including office administration, scientist, custodian, and sales manager. Most participants (82%) reported they had been suffering from cLBP symptoms for over one year.

**Table 1:** Participant characteristics prior to intervention (at baseline)

Demographic (***n*** = 11)	Median (Q1, Q3) or n (%)
Age (yrs.)	42 (32, 45)
Sex (% male)	6 (55)
Race (% white not Hispanic)	9 (82)
Employment Status (% full-time)	10 (91)
Income (% ≥ $100,000 yearly)	6 (55)
Education (% postgrad degree or higher)	4 (36)
Impact of cLBP (MDS)	23 (17, 27)
Depressive Symptoms (PHQ-9)	8 (6, 8)

MDS: Minimal Data Set for chronic low back pain; PHQ-9: Patient Health Questionnaire-9.

Table 2 provides information about participants’ changes in activity levels and habits across the intervention. During the intervention, on average, participants broke up their sitting time through accumulating 250+ steps per hour on 9.0 ± 1.8 h per day (based on data from the provided activity monitor). At the end of the intervention, participants reported sitting approximately two hours less than they reported sitting pre-intervention. ActivPAL-assessed behavior showed smaller changes, with participants sitting 36 min less and accumulating 966 more steps, on average, per day. However, these changes in activity levels varied greatly across participants (ranging from sitting ~ 40 more minutes per day to sitting ~ 2 h less per day). Data from the SRHI showed that frequently standing up and breaking up longer bouts of sedentary time felt more habitual post-intervention, while sitting when they could stand felt less habitual.

**Table 2:** Change in sitting habits and activity levels

Habit Development	Pre	Post	Change
Habit “Frequently standing up”	38 (28, 47)	49 (42, 58)	13 (21, 8)
Habit “Breaking up long bouts of sedentary time”	35 (28, 40)	45 (42, 63)	16 (17, 7)
Habit “Sitting when I could stand”	41 (36, 52)	38 (29, 42)	−12 (7, 22)
**Self-Reported Sitting Time**			
Occupational-related sitting (hr.)	4.8 (2, 7)	3.3 (2, 4.5)	−1.5 (−3, 0)
Number of breaks in work sitting time per day	4 (2.5, 6)	5 (3.5, 5)	0 (−1, 2)
Number of breaks in screen time per day	2 (1, 2.5)	2 (1, 3)	0 (−1, 2)
Weekday sitting (hr.)	11.6 (11.3, 13)	9.4 (7, 10)	−2.8 (−5.9, −1.2)
Weekend sitting (hr.)	11 (8, 13.5)	6.5 (6, 11.5)	−2 (−5, − 1.3)
Change in total sitting (hr.)	11.3 (10.6, 12.4)	9 (8.3, 10.4)	−2.6 (−5.5, −0.9)
**Monitor Assessed Sitting Time**			
AP standing time (hr.)	3.9 (3.7, 4.7)	4 (3.6, 6.5)	0.6 (−0.05, 1.6)
AP sitting time (hr.)	11.4 (10.2, 11.7)	11.2 (8.9, 11.5)	−0.5 (−1.2, 0.6)
AP time spent in sitting bouts > 30 (hr.)	5.9 (4.8, 6.4)	5.3 (4.1, 6.8)	−0.2 (−1.3, 0.9)
AP time spent sitting in bouts > 60 (hr.)	3 (2.7, 4)	2.9 (2, 3.4)	−0.1 (−1.5, 0.3)
Total number of steps per day	8429 (7598, 9740)	9182 (7589, 10,998)	555 (− 761, 2169)

Data are presented as median (Q1, Q3). Change scores are post-intervention values minus pre-intervention values. Abbreviations: hr. = hour; AP = activPAL.

### Key themes

Reported factors that were perceived to influence the ability to reduce or break up SED were thematized as positive or negative determinants. Negative determinants of reducing SED (i.e., perceived barriers that made is difficult to sit less) included environmental constraints, social norms and productivity. Positive determinants of reducing SED (i.e., perceived facilitators that made it easier to sit less) included habit development, self-monitoring tools, physical environment, and social accountability. These are each described in more detail below.

#### Negative determinants of SED

##### Environmental constraints

Eight participants reported that environmental constraints, and particularly those related to the workplace, made it challenging to reduce sitting. Commonly reported examples were work-related tasks that typically require sitting, such as computer work or lab procedures that necessitate benchwork. Many tasks are time-sensitive, so taking a break to walk was perceived as impractical or impossible. Necessary transportation and weather were also reported to influence sitting time. Specifically, longer car trips and colder weather made it challenging to breaking up prolonged SED and reduce overall sitting.

*“Most of it was just due to my requirements in the fact that a big portion of my job is computer based, so I'm either at home, in a home office sitting, or sitting in a pickup traveling … to an off-site meeting, so you know, unfortunately a big aspect of my job is sitting.”*

While taking breaks to walk was not perceived as feasible at work, using standing desks and backless chairs/stools were commonly reported techniques used during the intervention period to help break up and reduce sitting, despite environmental constraints. These ideas were coded as a positive determinant and are discussed below.

##### Social norms

Eight participants stated it was difficult to sit less during certain social activities in which others were seated, especially when work-related. Many participants reported feeling uncomfortable or ‘weird’ when going against what they perceived as usual, seated workplace behavior. Two participants described feeling disrespectful if standing during seated meetings or disruptive to others if attempting to stand to break up prolonged sitting.

*“We have trainings where it feels socially unacceptable to stand up when everyone else is sitting and paying attention. It felt just too uncomfortable to bring attention to myself I guess.”*

*“It’s kind of awkward if someone is standing in the back of the room, or the front of the room, or the side of the room when everyone else is sitting and talking”*

Participants reported that work-related social norms surrounding sitting would need to be changed to reduce the impact of this barrier. For smaller, informal meetings, participants indicated walking meetings and standing meetings would be beneficial.

*“We do have conference rooms that have taller desks in them, so if it’s a smaller meeting with just a small team, there were standing level tables that we could stand at it, so I would try to book those conference rooms and then just stand.”*

For larger meetings, leadership from the meeting hosts would be needed. Participants indicated they could sit less if leaders of the meeting would state that they encourage standing, provide space in the room for standing (e.g. standing tables behind seating), or incorporate standing or walking breaks during the meeting.

*“[If] whoever is running the meetings says, ‘Hey let’s take a five-minute break’. Whenever we get breaks, I take that as an opportunity to stand up and stretch and get water, so having the scheduled breaks when everybody is free [to] move around, feels less conspicuous.”*

##### Productivity

High concentration on work in combination with motivation to be productive was frequently reported as a barrier to reducing SED by eight participants. Participants reported ignoring prompts from the activity monitor to complete work tasks and/or not lose traction or progress on a task. Others stated they would lose track of time and not feel the monitor vibration because they were so focused.

*“Even though I know I can get up and get away from the computer its hard if I’m in the middle of something, and I just want to finish it and even if I get a reminder thing ‘you need to get up and move’, if I was in the middle of something I just want to finish it and I don't want to stop and come back, so that was probably the most difficult.”*

Participants suggested more salient reminders of SED would be necessary during times when they were extremely focused on work. Ideas like apps that lock work items until a break is taken, multi-media (e.g. audio and vibratory) reminders, and *‘chairs that poke you’* were suggested.

*“Maybe a Fitbit vibrating on your wrist isn’t enough and so you need something underneath you to poke you and get you up and moving.”*

Participants reported that changing work-related social norms, such as institution-wide walking breaks each hour, could be valuable as it would demonstrate higher-level support for taking activity breaks from work.

*“If I talk to my co-workers and just said ‘Hey, let's all help each other with this and set an alarm and then just remind each other to all go for a 10-minute walk or go upstairs and walk around for a minute or two and then come back down,’ and did it as a group effort thing, to remind each other.”*

#### Positive determinants of SED

##### Habit development

Education about habits and working on development of habits of sitting less was perceived as very influential in changing behavior by all eleven participants. Participants expressed that implementation of external cues, such as vibratory reminders from the activity monitor, writing ‘break’ notes in margins of work documents and carrying a water bottle, were noticeable reminders to sit less. External cues from the activity monitor provided tailored feedback on SED with reliable, consistent reminders/prompts that were perceived as important in generating strong impulses for action over time.

*“[The device] is a machine that doesn’t forget that you need to move. It’s going to give you that reminder consistently, and so I think that's the best way to form a habit is to be consistent*.”

Participants described how internal cues, like back pain, were helpful but less reliably prompted changes in behavior. Specifically, they indicated that back pain, as a cue, did not provide the consistency that external cues did and was more challenging to associate with sitting less. Further, internal cues may have been easier to dismiss, especially since cues like pain have been long associated with other coping strategies like ignoring the pain or resting.

*“I was better at listening to the outward cues than listening to the inward cues that my body is giving me. I feel like I could ignore those easier, the inward ones, just because I've been doing it for so long and ignoring them for so long.”*

*“I associate being sore or being in pain…with me needing to rest and so by the time those presented themselves I didn’t want to go and break up my daily routine."*

##### Self-monitoring tools

Self-monitoring tools and strategies were consistently reported as facilitators to behavior change by all eleven participants. Participants described that prior to enrollment in the study, they were uninformed regarding the health risks associated with SED and recommendations for sitting less. Additionally, they were largely unaware of their personal SED. During the intervention period, both sedentary idle alert prompts (e.g. obtain 250+ steps each hour) and physical activity data (e.g. steps per day) were reported as advantageous in working towards sitting less.

*“The Fitbit was a huge part, so at ten till the hour, it buzzed at you to say get up and move, so that's a nice reminder.”*

*“The movement reminders [were helpful] because once you start doing stuff, you don't always think about it so having [the monitor] was nice to remind you [that] you’ve been sitting for a long time [and] it's time to get up and move.”*

*“It was really nice to have that constant reminder both at work and then in the evening because you'll sit down and watch TV… so having that reminder to break it up is really really nice. You don't realize how inactive you are throughout the day until you actually start seeing the numbers.”*

Some participants self-initiated coping planning for situations they anticipated would make sitting less more challenging.

*“I made it a point when I write to keep the aims on the side [of a document] and in between aims, I take a break and go for a short walk for 5 minutes to gather my thoughts before I start working on the writing part. So not only is [it] helping with my movement requirements but it's also helping me refocus.”*

*“Just traveling from location to location, there are times you could just strategically stop in the middle of an hour or on the hour—just stop at a rest stop or stop at a gas station to move around.”*

##### Physical environment

Seven participants stated that having a physical environment supportive of standing was largely important to meeting their goals. Many reported using standing desks to alternate between sitting and standing throughout the workday. For those without standing desks, the use of a backless chair or stool was found to be helpful in breaking up prolonged sitting.

*“I actually was able to get a standing desk so I can sit and stand at my desk. So, I could easily just move my desk so I could stand during a meeting. I wasn't able to move anywhere but at least I wasn't sitting.”*

*“Something I did do in the lab, I changed my nice, cushy chair to a slightly more uncomfortable stool, where it's a little bit more comfortable to stand than it is to sit on it, and so that's really helped, just changing that out.”*

##### Social accountability

Accountability was reported as a positive determinant by ten participants. Having social support from family, co-workers, and the research team assisted in reducing SED. Other people’s awareness of their behavioral goals and support of attempts to change their sitting habits was important for developing new routines of sitting less.

*“Coworkers knew too [of my goal], so they knew that I need to get up and move and things like that too, so just kind of that awareness helped.”*

#### Thematic network mapping

**Fig. 1:**
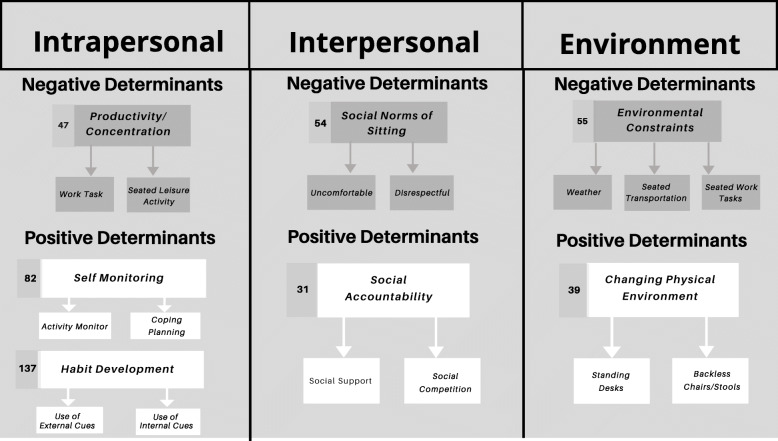
Network display of perceived determinants of reducing SED in context of an ecological model. Arrows away from 7 themes indicate codes that were prominent within each theme. Numbers to the left of each theme indicate total number of times the theme was coded across the 11 transcriptions and among coders

A network display of reported positive and negative determinants in the context of the ecological model of behavior change [25] is shown in Fig. 1. At the intrapersonal level, concentration/productivity were perceived barriers while self-monitoring and habit development were prominent facilitators of behavior change. Interpersonally, social norms of sitting were challenging to overcome. Having social accountability aided in accomplishing personal sitting goals and may be helpful for changing social norms. At the organizational level, environment constraints, primarily coming from work-related tasks, transportation, and the weather were barriers when attempting to sit less. Changing the physical environment by using standing desks and stools may help reduce the impact of this determinant.

## Discussion

This study explored perceptions of factors that influenced participants’ ability to reduce sitting after taking part in an intervention to reduce total and prolonged SED in 11 individuals with cLBP and elevated symptoms of depression. Overall, social (sitting norms and work expectations) and environmental factors were reported as prominent barriers for reducing SED, while intrapersonal factors (self-regulation, habit development) were perceived as being helpful in changing sitting habits and overcoming negative determinants.

Interestingly, physical pain and discomfort were not reported as barriers for reducing SED. This is in contrast to previous qualitative work. For example, studies have reported that pain and fatigue in older women [10], health-related problems in African American women [26], and physical health/injuries and fatigue in college-students [27] influenced sitting time. Our results showing a lack of influence of pain on perceptions of the ability to sit less are also in contrast to determinants for increasing physical activity, for which pain is a frequently cited barrier in this population [28]. While individuals may associate higher intensity activities with greater pain, our data suggest that they may have lower movement-related fear beliefs when focusing on reducing sitting, as opposed to increasing activity. Thus, a key finding from this study is that individuals with cLBP may perceive goals to reduce SED as easier and more feasible than goals targeting increases in physical activity when seeking to improve pain and/or general health.

Other factors that were perceived as influential are consistent with previous qualitative research on perspectives of reducing SED, with factors identified at intrapersonal, interpersonal, and organizational levels as shown in Fig. 1. For example, Hadgraft and colleagues (2016) explored perspectives of the feasibility and acceptability of strategies to reduce SED in office workers [29]. They reported key factors including the nature of work (e.g. sitting at computer, not wanting to lose concentration or productivity) and common structural (i.e. organizational) and social (i.e. interpersonal) environments were conducive for sitting, which aligns closely with the barriers our participants experienced. Participants in the study by Hadgraft et al. (2016) also reported changing the work place structured environment, changing social norms at work (e.g. walking meetings, in-person communications), or employing wearable fitness monitors as favored strategies for reducing SED. Qualitative studies in other populations, including older adults, African-American women, and college students, have also found the physical environment and social norms to be prominent factors in changing SED [10, 26, 27]. In support of these findings, a recent systematic review by Rawling and colleagues (2019) examining 30 studies, also concluded that there are important determinants across levels of the ecological model, with the most influential determinants falling under socio-cultural and environmental/organizational categories [30]. Similar to other populations, our sample of participants with cLBP reported being highly influenced by social and environmental factors indicating these factors as potentially high impact targets for future interventions targeting SED reductions.

Theory-based strategies are typically used to overcome common barriers to behavior change and participants indicated several of these strategies were helpful in sitting less. The intervention in this study employed strategies from the Self-Determination Theory via Motivational Interviewing (e.g. intrinsic motivation), habit theory (prompts/cues), and mHealth (e.g. self-monitoring from activity monitor) [31–33]. While care was taken to not explicitly include motivational factors as part of the qualitative interview, constructs of mHealth, habit theory, and restructuring the physical environment were frequently reported as helpful for reducing SED. This is consistent with data from a review of behavior change theories for SED by Gardener and colleagues (2016) that found self-monitoring of behavior, problem solving, and restructuring social or physical environments to be ‘very promising’ behavior change techniques for reducing SED [34]. Gardner et al. reported that ‘very promising’ interventions also utilized prompts/cues and habit formation strategies. Further, a meta-analysis of randomized controlled trials that implemented behavior change strategies also found that interventions targeting reductions in SED using mobile technology yielded a mean reduction of 41 min of SED per day, with prompts/cues, social support, and self-monitoring of behavior reported as important strategies [35]. The use of habit formation, an activity monitor for self-regulation, and restructuring the environment not only demonstrate promising effects in reducing SED but were also reported as helpful by participants with cLBP in this study.

Perspectives on the utility of habit theory for reducing SED may be especially important. Each participant reported that discussing and actively working on changing sitting habits was an important contributor to their success in reducing SED. Most participants felt they had adopted a strong habit of receiving an external cue from the activity monitor and standing or walking for a quick break. In other words, participants in this study expressed that they felt strong impulses toward action when encountering the cue. As noted above, the situations that were most difficult in following through with the habit routine were when concentrated on seated work and in seated, social situations in the workplace. These situations are examples of the impulse-behavior combination, “No behavior due to impulse inhibition” that Gardner (2015) proposed [36]. In other words, participants experienced strong impulses toward standing/breaking sitting from the external cue from the activity monitor, but the impulse was inhibited due to stronger social and environmental constraints. Thus, other strategies for these specific times are needed to help individuals develop sustainable habits surrounding sitting less and using external cues effectively. Coping planning or developing situation-specific plans to overcome anticipated barriers, may mitigate the influence of social norms and environmental constraints [37]. Specifically, coping planning may aid in making standing and/or taking breaks from sitting more salient options that require less cognitive effort. In this study, participants reported that planning out breaks each hour during long drives and structuring intentional breaks into work tasks before beginning them aided in following through with the behavior. Preparing for anticipated barriers with detailed coping plans tailored to participants may be key in overcoming social and environmental determinants.

Habit development was reported by participants to be highly reliant on external cues. External cues (e.g. prompts from the activity monitor) were coded 61 times across the 11 transcripts as a facilitator of behavior change. Many participants admitted they were largely unaware of the amount of time they spent sitting and/or unaware of the associations sedentary time has with health outcomes prior to enrollment in the study. Therefore, if unaware and unable to recognize prolonged sedentary time, internal cues and associated rewards may not be salient enough to promote habit development and behavior initiation. Initial, weak habits reliant on internal cues may not translate into behavior maintenance, as the cue that triggers the habit may not be sufficient and/or the behavior may not be repeatedly performed. Having an external mechanism to help self-regulate the behavior (e.g. mHealth, activity monitors) may help initiate a new behavior by tracking and regularly prompting the behavior, and potentially lead to stronger, more sustainable habits. A downfall of wearable technology for habit development is that maintenance of the behavior may be directly related to maintenance of the device; thus, a lost, broken, or forgotten monitor could interfere with behavior change [38]. However, if the activity monitor is consistently used during behavior initiation, new habits that are not reliant on the external monitor cues may also emerge (e.g., habitually planning breaks prior to beginning tasks, reserving standing meeting rooms, filling a water bottle each hour). How external cues from activity monitors facilitate the development of other external and internal cues not reliant on the monitor warrants investigation.

### Strengths and limitations

This study provides key information regarding determinants of sedentary time in individuals with cLBP, which has not been explored previously. Participants were encouraged to openly reflect and report on factors that influenced their ability to reduce SED. Further, these data provide participant perspectives *following* an intervention that focused on attempting to change SED. Thus, participants may have a clearer idea of factors that influence change than individuals who have not recently undergone such an experience. In addition, the research team implemented several strategies during data collection and analysis to ensure trustworthiness of the qualitative data, including thick and thin descriptions of the data, triangulation of coding, member checking, incorporating some mixed-methods data, and demonstrating reflexivity (e.g. discussing potential biases) in reporting [23].

Aside from stated strengths, there are limitations. There may be inherent biases in the reporting of positive determinants, as participants were educated about habit development, instructed to use their activity monitors during the intervention period, and subsequently asked about the influence these factors had on their ability to sit less. Further, the theoretical strategies from SDT were not assessed, nor was the motivational interviewing fidelity, so little is known about the potential influence (or lack of influence) of the health coaching sessions, although both sessions for each participant were performed by the same health coach. It is also plausible that other theoretical constructs that may have influenced behavior change may not have been reported because they were not probed for in the semi-structured interview (see Additional File 1 for interview questions). Moreover, although participants were asked to respond candidly during the qualitative interview, they may have responded in a way they perceived as favorable to the research team, as interviews for this study were performed by the health coach who also delivered the in-person intervention session and performed the 4-week phone call. Lastly, the sample was small and relatively homogenous, primarily consisting of white, college-educated adults, with relatively high incomes. Nonetheless, their duration and frequency of pain symptoms was representative of cLBP patients in general.

## Conclusion

In this sample of adults with cLBP and at least mild symptoms of depression, barriers to reducing sitting were consistent with previous findings in healthy populations and included environmental constraints, social norms, and productivity. Conversely, developing new habits around sitting less, implementing self-monitoring strategies, restructuring the environment, and having social accountability made sitting less feel easier in individuals with cLBP. Accounting for these factors, workplace interventions may be more effective if they consider the use of standing desks, standing meeting rooms, and incorporating institution-wide standing/walking breaks to overcome work-related factors that hinder one’s ability to reduce SED. Sedentary interventions would also likely benefit from the use of activity monitors to improve self-monitoring of sitting time and to serve as an external, real-time reminder to sit less. Importantly, education about how the reminders can serve as an external cues may be key in developing new sitting habits. Lastly, developing tailored coping plans for overcoming social sitting norms and inflexible environmental constraints may be particularly helpful for reducing SED.

## Supplementary Information

**Additional file 1.** Semi-Structured Qualitative Interview over Sedentary Intervention. The semi-strucutred interview (i.e. interview guide) developed for and used in this study.

**Additional file 2.** Coding Research Team Personal Characteristics. Description of personal characterisitcs of the research team members who completed the coding procedures and qualitative analysis.

**Additional file 3.** Deductive Codebook. Table of categories, abbreviations, and definitions of codes used in analysis.

## Data Availability

The datasets generated and/or analyzed during the current study are not publicly available due to ethical requirements but are available from the corresponding author on reasonable request.

## Abbreviations

SED: sedentary time
cLBP: chronic low back pain
SRHI: self-reported habit index
