# Supplementary material for: A qualitative analysis of barriers and facilitators to reducing sedentary time in adults with chronic low back pain
Source: BMC Public Health. 2021 Jan 26;21:215. doi: 10.1186/s12889-021-10238-5 (PMC7836448; doi:10.1186/s12889-021-10238-5)
Supplement: Supplementary file 1 — Additional file 1. Semi-Structured Qualitative Interview over Sedentary Intervention. The semi-strucutred interview (i.e. interview guide) developed for and used in this study. [file 12889_2021_10238_MOESM1_ESM.docx]

**Semi-Structured Qualitative Interview over Sedentary Intervention**

After completing the 8-week intervention to reduce your sedentary time, we’d like to ask a few questions about your experience. Your responses are extremely valuable in helping us and other researchers develop similar interventions in the future. With that in mind, it is important that you are as honest and candid as possible.

First, thinking about your sedentary time over the 8-week period, what were the primary reasons you found yourself sitting for long periods of time?

- In which of these situations were you able to decrease or break up your sitting time?
  - What steps did you take to help you reduce your sitting during each of these situations?
  - Can you identify any factors that made this easier for you?
- Again, thinking of those situations where you were sitting for long periods of time, when was it most challenging to decrease or break up your sitting?
  - For those times when it could have been possible to decrease your sitting, tell me about how you tried to sit less during these times?
  - Can you identify any factors that made these times more difficult for you to reduce your sitting?
  - What do you think you would need in order to sit less during these times?

Now I’d like to talk about your experience with the Fitbit and how it influenced your ability to decrease or break up your sitting.

Overall, describe your experience using the Fitbit to help you reduce your sedentary time.

In terms of receiving prompts, how helpful were the reminders and feedback you received from the Fitbit and associated application?

- Would other prompts be more beneficial?
- If so, which types? (e.g. emails, texts, alarms)
- How frequently?

As part of the intervention, we also discussed sitting as a habit and strategies for forming new habits. How well do you feel you were able to build new habits?

- Overall, did thinking about habits help you decrease or break-up your sitting time?
- We talked about inward cues like pain or an emotion and outward cues like the fitbit or pillows on the couch blocking your seat. Thinking about both of these types of cues, which were most helpful in working toward developing a new habit?
- What cues were least helpful in working toward developing new habits?

Aside from the Fitbit, were there any other strategies or tools that helped you or reminded you to sit less?

- If you were to design an intervention with unlimited resources to help you sit less, what would it look like?
