# Supplementary material for: A qualitative analysis of barriers and facilitators to reducing sedentary time in adults with chronic low back pain
Source: BMC Public Health. 2021 Jan 26;21:215. doi: 10.1186/s12889-021-10238-5 (PMC7836448; doi:10.1186/s12889-021-10238-5)
Supplement: Supplementary file 2 — Additional file 2. Coding Research Team Personal Characteristics. Description of personal characterisitcs of the research team members who completed the coding procedures and qualitative analysis. [file 12889_2021_10238_MOESM2_ESM.docx]

| **Coding Research Team Personal Characteristics (JL, KS, GC)** | |
| --- | --- |
| **Sex** | 2 female, 1 male |
| **Education Level** | 2 PhD, 1 MS |
| **Occupation (i.e. time of study)** | 2 Graduate Research and Teaching Assistant  1 Assistant Professor of Kinesiology |
| **Specialty Area** | 2 Exercise Psychology, 1 Health Promotion |
| **Experience and Training** | All coders completed graduate level courses on determinants and theories of behavior change and statistical analyses, as well as reviewed Miles et al. 2019 for additional information on qualitative analysis [20] |
