# Supplementary material for: A qualitative analysis of barriers and facilitators to reducing sedentary time in adults with chronic low back pain
Source: BMC Public Health. 2021 Jan 26;21:215. doi: 10.1186/s12889-021-10238-5 (PMC7836448; doi:10.1186/s12889-021-10238-5)
Supplement: Supplementary file 3 — Additional file 3. Deductive Codebook. Table of categories, abbreviations, and definitions of codes used in analysis. [file 12889_2021_10238_MOESM3_ESM.docx]

| **Deductive Codebook** | | |
| --- | --- | --- |
| **Category: Negative Determinants, Abbreviation, Definition** | | |
| Occupational: Constraints | N_O_CONS | Job tasks require participant to be seated (e.g. working on the computer at seated desk, working on bench in lab) |
| Occupational: Productivity/Concentration | N_O_PROD | Standing or taking breaks disrupts concentration on work or reduces productivity |
| Social Norms: Feels Uncomfortable | N_SN_UNCO | Standing feels uncomfortable because other people are seated or do not take breaks |
| Social Norms: Disrespectful | N_SN_DISR | Participant indicates they feel disrespectful or impolite to others they are conversing with |
| Physical Discomfort | N_PHYSDIS | Participants indicates they did not stand or take breaks due to physical discomfort, such as pain, fatigue, or discomfort |
| Social Norms: Preferences | N_SN_PREF | Lack of motivation due because of enjoyment/pleasure from seated activity (e.g. movie, game, knitting); they prefer to sit during this activity |
| Access to Resources | N_ACCRES | Did not stand/break up because lack of resources, such as a standing desk to work |
| Time | N_TIME | Unaware of amount of SED time (e.g. lost track of time or did not even feel monitor prompt) |
| Habit | N_HB | Participant indicates a habit of sitting. They may be largely unaware of sitting time and/or sit due to routine. |
| Weather | N_WTHR | Weather (e.g. cold outside, extreme heat) provides barrier for reducing sitting. |
| **Category: Positive Determinants** | | |
| Accountability | P_ACCT | Accountability from research team, co-workers and/or family |
| Activity Tracker: SED Prompts | P_FB_SB | Activity monitor for reminders when sedentary for prolonged time |
| Activity Tracker: PA Goals | P_FB_PA | Activity monitor for physical activity related data (e.g. steps per day) |
| Activity Tracker: Competition | P_FB_COMP | Using tracker to compete against self or others with daily SED or PA goals |
| Education | P_EDU | Education about risk factors associated with sitting and/or about viewing data on individual SED levels (e.g. activPAL data) |
| Social Norms: Change in Work Norms | P_SN_WKCH | Implementation of new social norms at work (e.g. walking meetings, break each hour to walk, team competitions) |
| Occupation: Change in Leadership | P_O_LEAD | Implementation of breaks in meetings/trainings by leaders/speakers |
| Physical Environment | P_ENVR | Changing environment (e.g. standing desk, stools instead of chairs) |
| Habit Development | P_HB_DEV | Development of habits for sitting less; feels natural to take breaks |
| Habit: External Cue | P_HB_EX | Attention to habits using external cues, such as FB, water bottle, or notes |
| Habit: Internal Cue | P_HB_IN | Attention to habits using internal cues, such as noticing cLBP or fatigue |
